# Ultrasound-guided thoracic paravertebral injection of dexamethasone palmitate combined with ropivacaine for the treatment of thoracic herpes zoster-related pain: protocol for a prospective, randomized controlled, single-center study

**DOI:** 10.3389/fphar.2024.1470772

**Published:** 2025-01-07

**Authors:** Liu Wang, Shengrong Xu, Zongbin Jiang, Ruilin He

**Affiliations:** Department of Pain Medicine, The Second Affiliated Hospital of Guangxi Medical University, Nanning, Guangxi, China

**Keywords:** herpes zoster pain, pain management, paravertebral injection, dexamethasone palmitate, ropivacaine

## Abstract

**Background:**

Herpes zoster (HZ) patients often experience herpes zoster-associated pain (ZAP). Thoracic paravertebral nerve block has been proven effective in relieving ZAP and reducing the incidence of postherpetic neuralgia (PHN). Compared to dexamethasone, dexamethasone palmitate (DXP) has stronger anti-inflammatory effects, a longer duration of action, and fewer adverse reactions. This study evaluates the efficacy and safety of ultrasound-guided thoracic paravertebral injection of DXP combined with ropivacaine for treating thoracic ZAP, compared to traditional famciclovir therapy.

**Methods:**

This prospective, randomized, controlled, open-label, endpoint-blinded, single-center trial will recruit 254 patients with ZAP. Patients will be randomly assigned in a 1:1 ratio to the intervention group (thoracic paravertebral injections of DXP combined with ropivacaine and antiviral therapy) or the control group (antiviral therapy). Assessments will include pain intensity, quality of life, sleep quality, inflammatory markers, and adverse events.

**Ethics and registration:**

This study strictly adheres to the 2013 SPIRIT Statement and the Declaration of Helsinki and has been approved by the Ethics Committee of the Second Affiliated Hospital of Guangxi Medical University (Approval Number: 2024-KY(0505)). This clinical trial is registered on the Chinese Clinical Trial Registry platform (ChiCTR) at https://www.chictr.org.cn/index.html (ChiCTR2400087273), registered on 2024-07-24. The results will be disseminated through scientific journals and conferences, aiming to provide evidence supporting the global management of ZAP. The study is expected to start on 1 August 2024, and continue until 31 July 2027.

## Introduction

In adults, the reactivation of varicella-zoster virus (VZV), which lies dormant in the dorsal root or trigeminal ganglia, typically results in herpes zoster (HZ). This condition is more common in individuals over the age of 50 (Sun et al., 2021; Cohen, 2013). Due to the neural damage and acute neuritis caused by VZV, patients experience persistent and severe pain, referred to as herpes zoster-associated pain (ZAP), which can become severe during the acute phase of the disease and often progresses to postherpetic neuralgia (PHN) if it persists beyond 90 days, posing significant treatment challenges (Ahronowitz and Fox, 2018; Ma et al., 2022; Rowbotham and Petersen, 2001).

Despite the widespread use of antiviral agents, anticonvulsants, antidepressants, and opioid analgesics, a subset of patients may still require additional therapeutic interventions to achieve adequate symptom control (Patil et al., 2022). This is particularly pertinent for individuals presenting with higher initial pain scores, advanced age, or comorbid conditions, as they remain at an elevated risk for developing PHN (Hashizume et al., 2022; Zhou et al., 2021; Wang et al., 2019). PHN significantly impacts patients' daily lives, leading to anxiety, depression, and sleep disturbances, making the prevention of PHN crucial for HZ patients (Wang et al., 2019). The side effects of medications, such as dizziness and drowsiness from gabapentin or pregabalin, and gastrointestinal issues from opioids, further complicate management strategies (Wang et al., 2023). Therefore, it is essential to minimize ZAP and prevent the occurrence of PHN.

Neuropathic pain is characterized by alterations in ion channels, activation of immune cells, and the involvement of neuroglial mediators. Among the commonly used medications, sodium channel blockers play a crucial role in managing this condition (Wang et al., 2021a). Ropivacaine, a potent sodium channel blocker, achieves its analgesic effect by reversibly inhibiting sodium ion influx, thereby blocking nerve impulse conduction. It is noted for its prolonged duration of action compared to lidocaine and is widely recognized for its safety due to its lipophilic properties, which confer reduced toxicity to both the cardiovascular and central nervous systems (Kaufman et al., 2005). A 0.2% concentration of ropivacaine is effective in providing analgesia with minimal motor impairment, making it suitable for epidural analgesia (Dresner et al., 2000).

Corticosteroids are frequently employed for postoperative pain relief in various surgical procedures, yielding favorable outcomes (Bai et al., 2020). Dexamethasone, a potent corticosteroid with an extended duration of action, has been demonstrated to provide effective adjunctive analgesia in procedures such as total knee arthroplasty (El-Boghdadly et al., 2021) and tonsillectomy (Basuni et al., 2013), as well as prolonging the duration of nerve blocks (Hauritz et al., 2018). It is commonly used as an adjunct for pain relief (Chong et al., 2017). Intrathecal steroid injections or nerve blocks are methods for effectively preventing and treating PHN (Wang et al., 2009). Ultrasound-guided thoracic paravertebral block (TPVB) is effective for ZAP and reducing the incidence of PHN (Ma et al., 2022; Abdelwahab et al., 2022; Deng et al., 2023; Kim et al., 2017).

Dexamethasone palmitate (DXP) is a liposomal formulation of dexamethasone, which exhibits superior anti-inflammatory efficacy, prolonged action, and reduced side effects (Lorscheider et al., 2019). DXP has been effectively utilized for pain management in conditions such as musculoskeletal disorders (Lee et al., 2022), rheumatoid arthritis (Lorscheider et al., 2019), and in video-assisted thoracoscopic surgery (Hui et al., 2023). While particulate steroids in epidural injections have been implicated in postoperative embolic events (Malhotra et al., 2009), mixing ropivacaine with dexamethasone sodium phosphate does not result in significant particulate formation, thereby enhancing safety (Wang et al., 2021b). Consequently, the combination of DXP and ropivacaine is considered both safe and effective.

A previous prospective study involving 10 patients with ZAP demonstrated the safety and efficacy of thoracic paravertebral injections of a single low-dose mixture of DXP combined with ropivacaine. However, this study was constrained by a limited range of outcome measures and the absence of long-term follow-up, hindering a comprehensive assessment of the long-term safety and efficacy of this therapeutic approach (Pu et al., 2022).

To rigorously evaluate the effectiveness of DXP combined with ropivacaine in treating ZAP and preventing PHN, a larger-scale, prospective, randomized, single-center study is warranted. Given the prolonged duration of action of ropivacaine and the enhanced anti-inflammatory efficacy, extended duration, and reduced side effects of DXP (Lorscheider et al., 2019), we hypothesize that a single injection of DXP combined with ropivacaine will reduce the incidence of PHN and effectively alleviate ZAP. Only patients whose NRS-11 score remains ≥4 after 2 weeks of intervention will receive an additional ultrasound-guided thoracic paravertebral injection.

### Objectives

The primary objective of this trial is to compare the efficacy of DXP combined with ropivacaine versus antiviral treatment alone in preventing ZAP from progressing to PHN within 12 months of follow-up, with the primary evaluation metric being the incidence rate of PHN, defined as a Numeric Rating Scale (NRS-11) score of ≥4 3 months after rash onset.

Secondary objectives include assessing the reduction in pain intensity, improvement in sleep quality (using the Pittsburgh Sleep Quality Index), enhancement of quality of life (using the Short Form Health Survey SF-12), decrease in weekly analgesic consumption, and changes in levels of inflammatory markers (C-Reactive Protein (CRP), Tumor Necrosis Factor-alpha (TNF-α), and Interleukin-6 (IL-6)).

## Methods

### Patient and public participation

Patients and/or the public did not participate in any stage of the design, execution, reporting, or distribution of the plans of this study.

### Study design and participants

This study is a prospective, randomized, open-label, endpoint-blind, single-center trial. The trial aims to recruit 254 patients with ZAP from the Second Affiliated Hospital of Guangxi Medical University. Participants will be randomly assigned in a 1:1 ratio to the intervention group or the control group. The intervention group will receive DXP with ropivacaine via thoracic paravertebral injection in addition to antiviral therapy, while the control group will receive antiviral therapy alone.

**FIGURE 1:**
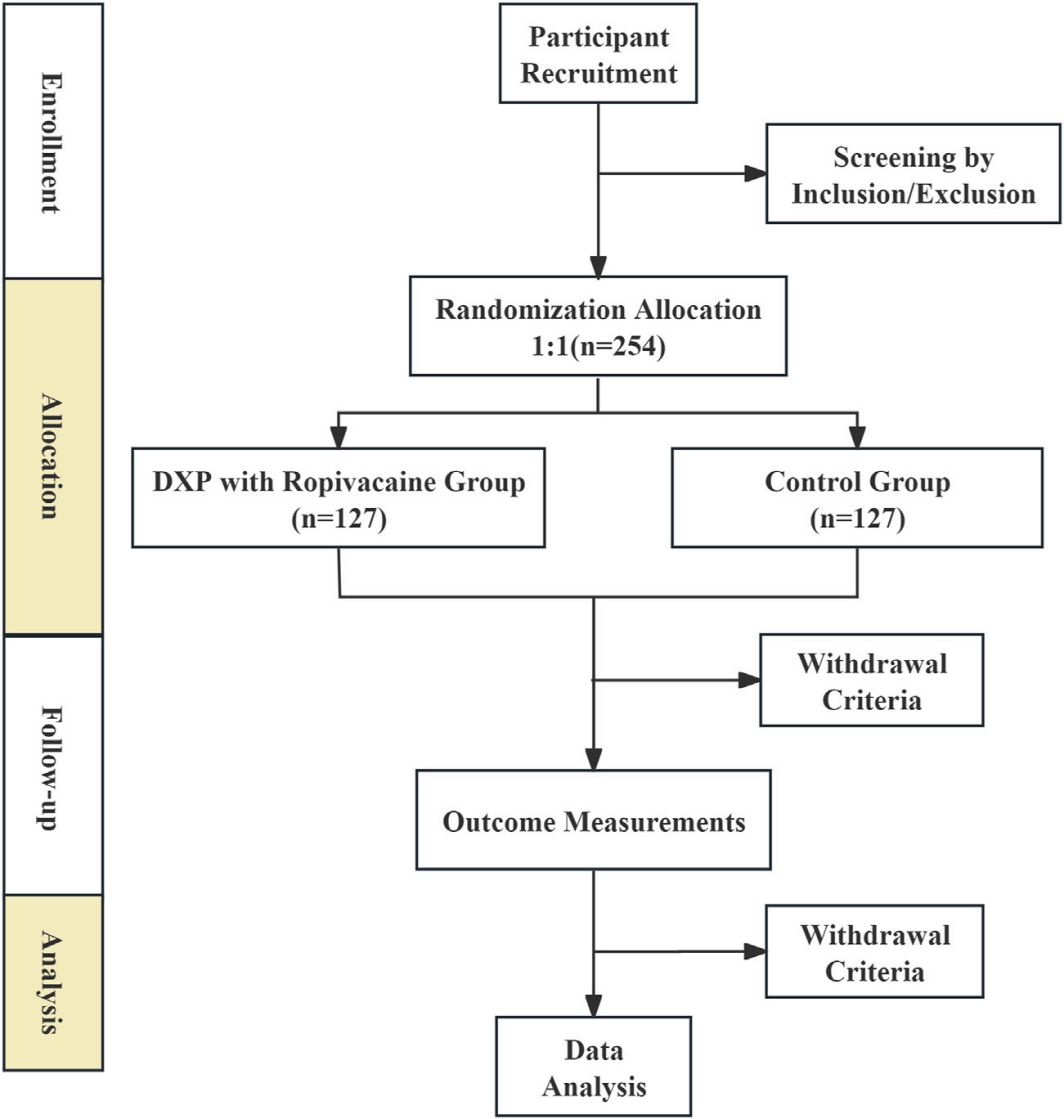
Trials flow diagram of the trial.

Before the study begins, all researchers will undergo standardized training covering the trial content, treatment strategies, methods, evaluation, and quality control, and they must pass an assessment. All patients will undergo a detailed explanation and informed consent process before enrollment and will receive comprehensive evaluations at baseline and during follow-up, including assessments of pain intensity, quality of life, sleep quality, and adverse events. The patient flowchart of this study is shown in Figure 1. This protocol conforms to the Standard Protocol Items: Recommendations for Interventional Trials checklist (Chan et al., 2013).

### Inclusion criteria

(1) Age ≥50 years with a diagnosis of thoracic HZ.
(2) Disease duration ≤2 weeks.
(3) Baseline pain intensity with an NRS-11 ≥ 4 points (Baseline pain intensity is the mean pain intensity score within 24 h before randomization).
(4) Patients experiencing a first episode of HZ who have not received any other interventions.

### Exclusion criteria

(1) Special types of HZ infection: Patients with disseminated HZ or HZ at other locations.
(2) Puncture risks: Patients with systemic infections, coagulation dysfunction, ongoing anticoagulant/antiplatelet therapy, infections at the puncture site, or thoracic spine abnormalities affecting ultrasound guidance.
(3) Special populations: Pregnant or lactating women, individuals with immunodeficiency, severe cardiovascular, cerebrovascular, respiratory diseases, or other serious systemic conditions that impede treatment cooperation, and patients with mental illness.
(4) Medication allergies: Patients with allergies to treatment-related drugs (e.g., steroid medications, famciclovir, gabapentin, pregabalin, Nonsteroidal Anti-Inflammatory Drugs (NSAIDs), tramadol) or contraindications for nerve block therapy.

### Withdrawal criteria

(1) Health Changes: Participants developing additional health issues, complications, or significant physiological changes that render them unsuitable for continued participation will be classified as dropouts.
(2) Adverse Events: Participants experiencing serious or critical adverse events, which, upon our research team’s assessment, necessitate discontinuation, will be withdrawn from the study.
(3) Protocol Violations: Participants who self-administer non-protocol drugs or undergo external treatments will be considered in breach of the protocol and will be excluded.
(4) Voluntary Withdrawal: Participants may opt to withdraw for personal reasons or may be lost to follow-up during the concluding phase of the study.

### Recruitment

Trained researchers will determine eligible patients based on the inclusion and exclusion criteria mentioned above. If patients meet the eligibility criteria, researchers will explain the study’s purpose, interventions, potential benefits, possible risks, and countermeasures to the participants. Each subject will have at least 1 h to ask questions about the study and decide whether to participate. Then, subjects will sign a written informed consent form and have the right to withdraw from the study at any time. The confidentiality of participants' records will be protected. All participants will be asked to allow the research team to share relevant data. If participants withdraw from the study, they will also be asked if they consent to the use of their data.

### Randomization and blinding

#### Randomization method

Participants will be randomly assigned in a 1:1 ratio to receive either antiviral treatment combined with DXP and ropivacaine or antiviral treatment alone, using a computer-generated random sequence created with IBM SPSS Statistics 25.0. The sequence will be stored in numbered opaque envelopes to ensure allocation concealment. The efficacy and safety assessments will be conducted by independent evaluators who are blinded to the group assignments.

#### Blinding method

Due to the nature of the interventions (nerve block vs oral antiviral medication), blinding of patients and treating physicians is not feasible. However, all medication packaging will be standardized by an independent pharmacy service to minimize bias related to treatment allocation guessing. Endpoint assessments for efficacy and safety will be conducted by a dedicated team of clinicians who are blinded to treatment allocation. This team will not be involved in the daily management of the participants.

#### Data handling and analysis blinding

Data collection and entry will be handled by an independent data management team. Statisticians performing the data analysis will remain blinded to treatment group allocations until the database is locked.

### Interventions

#### Control group

Patients in the control group will receive standard antiviral therapy with famciclovir 500 mg, administered three times daily for 7 days, immediately upon recruitment during the viral disease onset (Chen et al., 2014).

#### Intervention group

Patients in the intervention group will receive the same antiviral therapy as the control group. Additionally, they will undergo ultrasound-guided thoracic paravertebral injections. If the patient’s NRS-11 score remains ≥4 2 weeks after the initial intervention, they will receive an additional ultrasound-guided thoracic paravertebral injection. A 5 mL mixture consisting of 0.2% ropivacaine and 4 mg DXP will be injected at each thoracic paravertebral site corresponding to the affected nerves.

We adopted an ultrasound-guided approach for thoracic paravertebral nerve block, as detailed in Pu et al. (2022). The procedure involves a four-step confirmation of the thoracic segment, followed by an in-plane puncture technique. The midpoint of the thoracic inferior articular process and parietal pleura serves as the puncture endpoint. Real-time ultrasound imaging facilitates the accurate placement of the needle tip. A mixture of local anesthetics and corticosteroids is administered for effective neuropathic pain management.

All patients will receive standard pain management, including anticonvulsants, antidepressants, or opioid analgesics, if their NRS-11 score remains ≥4 after treatment (Valladales-Restrepo et al., 2023).

#### Study outcomes

The baseline demographics characteristics of the patients will be collected through searches of the institution’s electronic medical records or direct questioning of the participants. Data collected from the electronic medical records will include gender, age, BMI affected nerves, average weekly consumption of analgesics and levels of inflammatory markers. Data obtained directly from the patients will include pain scores (NRS-11 scores), the Pittsburgh Sleep Quality Index (PSQI), quality of life score, and pain characteristics for comparison with post-intervention outcomes. All patients will undergo systematic follow-up after receiving treatment. The follow-up schedule, outlined in Table 1, is designed to comprehensively assess the long-term outcomes of the treatment. Patients will be followed up at 1, 3, 6, and 12 months post-treatment. These time points were selected based on the natural course of herpes zoster-associated pain and the development of PHN. PHN is typically defined as pain persisting beyond 3 months after the onset of the rash, hence the inclusion of the 3-month mark as a primary time point for assessing PHN incidence (Ahronowitz and Fox, 2018; Ma et al., 2022; Rowbotham and Petersen, 2001). The 1-month and 6-month follow-ups allow for the assessment of intermediate outcomes, such as pain intensity, sleep quality, and inflammatory markers, while the 12-month follow-up provides data on the long-term efficacy and safety of the intervention. The follow-up assessments include the following.

**TABLE 1:** Time schedule of participant enrolment, allocation and assessment.

	Enrolment	Allocation	Post-allocation											Close out
Time point	D0	D0	D1	D2	D3	D4	D5	D6	D7	D14	M1	M3	M6	M12
Enrolment														
Eligibility screening	√													
Informed consent	√													
Allocation		√												
Intervention														
Antiviral therapy			√	√	√	√	√	√	√					
Thoracic Paravertebral Nerve Block Treatment			√							*				
Assessment														
Demographics	√													
Incidence rate of PHN												√	√	√
NRS-11	√	√	√	√	√	√	√	√	√	√	√	√	√	√
Skin lesions	√	√	√	√	√	√	√	√	√	√	√	√	√	√
Pain characteristics and	√	√	√	√	√	√	√	√	√	√	√	√	√	√
SF-12		√							√			√	√	√
PSQI		√							√			√	√	√
Levels of inflammatory markers		√							√			√	√	√
Consumption of analgesics			√	√	√	√	√	√	√	√	√	√	√	√
Adverse events			√	√	√	√	√	√	√	√	√	√	√	√

*If NRS-11 ≥ 4 at D14, a second TPVB, injection may be performed.

PHN: postherpetic neuralgia; NRS-11: The Numeric Rating Scale; SF-12: 12-item Short-Form Health Survey; PSQI: pittsburgh sleep quality index; D: day; W: week; M: month.

#### Primary outcome

Incidence Rate of PHN: PHN is defined as a condition characterized by pain in the dermatomal area that persists for a duration exceeding 3 months following the resolution of the herpetic rash (Kost and Straus, 1996). The incidence rate of PHN will be precisely determined by assessing the percentage of patients who exhibit an NRS-11 score ≥4 during scheduled follow-up visits at 3, 6, and 12 months (patient-reported).

#### Secondary outcomes

(1) Pain Intensity: This parameter is quantified using the NRS-11, which provides a spectrum ranging from 0, signifying an absence of pain, to 10, indicating the most severe pain conceivable (patient-reported).
(2) Sleep Quality: PSQI serves as the instrument to assess the quality of sleep among patients (Buysse et al., 1989) (patient-reported).
(3) Quality of Life Score: Patient wellbeing is evaluated using the SF-12, a concise 12-item questionnaire designed to gauge the multifaceted aspects of health-related quality of life (Ware et al., 1996) (patient-reported).
(4) Average Weekly Consumption of Analgesics: This metric tracks the mean quantity of each pain-relieving medication consumed on a weekly basis (investigator-reported, based on medical records).
(5) Pain Characteristics: Presence of accompanying symptoms such as itching, sensory abnormalities, and hyperalgesia (patient-reported).
(6) Levels of Inflammatory Markers: Changes in the levels of inflammatory markers post-treatment, such as CRP, TNF-α, and IL-6 (investigator-reported, based on laboratory tests).
(7) Skin lesions: Including the number of days from the initial onset of the rash to its resolution, as well as the largest area of the skin lesion.

#### Safety evaluation

Adverse events (AEs) are defined as any unfavorable medical occurrences during the study, irrespective of their relation to the study medication or interventions. All AEs will be meticulously documented and promptly treated. The correlation of AEs with the study interventions will be thoroughly evaluated and reported to the Institutional Review Board (IRB). Study-related AEs will be treated free of charge until recovery. The study will be terminated immediately if any severe AEs result in extended hospitalization or death. The IRB will conduct regular checks on the trial.

### Data collection and management

To ensure the accuracy and completeness of the study data, the following data collection and management strategies will be employed.

(1) Electronic Case Report Form (eCRF Forms): The eCRF forms will serve as the primary data collection tools for this study, containing clear instructions and annotations. They will be designed to include range and format checks for data entry to minimize errors. Data collection and entry will be handled by two members of the research team, who will not be involved in data analysis, and will perform double-checking. Regular quality control checks will be conducted to ensure data integrity and accuracy. Data analysts will use a blinded dataset for analysis, with blinding maintained until the database is locked.
(2) Data Isolation and Confidentiality: Records containing participant names or other identifiable information (e.g., informed consent forms and contact information sheets) will be stored separately from the study data records. Study data will be identified only by participant number and initials to protect privacy.
(3) Data Access Control: Data will be stored in password-protected computer systems. Only the Principal Investigator (PI) and authorized research team members will have access to files related to personal data, with all access activities recorded to prevent unauthorized access.
(4) Database Management: The final database will be managed by the statistician for subsequent statistical analysis. Before analysis, the data management team will clean and anonymize the data to remove any information that might identify participants.
(5) Data Monitoring Committee (DMC) Role: The DMC will periodically review the database to ensure data quality and consistency. The DMC will review the database at key study milestones (after enrolling 30%, 50%, 80%, and 100% of participants) to check for missing, suspicious, and inconsistent data. The DMC will also receive interim results and regularly review safety reports to assess the strength of the evidence and the absolute degree and severity of any safety signals.
(6) Special Permissions: Access to the database for personal information retrieval requires special permission, which must be granted by the PI and the DMC. Apart from the PI and the statistician, no one else will have access to the database.

### Sample size calculation

Based on previous research data, the incidence of PHN in patients over 50 years old who receive standard antiviral treatment for HZ is 34.8%, while the incidence of PHN after repeated TPVB injections with lidocaine and triamcinolone is 16.3% (Ma et al., 2022). We predict a similar incidence rate in this study, based on the comparable treatment regimen and patient population. Sample size calculation is performed using PASS V.15 software (NCSS, Kaysville, UT, United States) with α = 0.05, β = 0.1, and power = 90%. Considering a dropout rate of 10%, we plan to recruit 127 patients per group, totaling 254 patients, with a 1:1 randomization ratio.

### Handling bias and missing data

Multiple imputation will be used to address missing data. For outliers, the decision to correct, remove, or apply data transformation methods will be based on the source and nature of the outliers to mitigate their impact. We will conduct sensitivity analyses to assess the impact of missing data. Potential sources of bias will be addressed through thorough data monitoring and regular audits by the DMC.

### Statistical analysis

Data analysis will be conducted using R 4.3.0 software. Both intention-to-treat (ITT) and per-protocol (PP) analyses will be performed. The ITT analysis will include all randomized participants, regardless of adherence to the protocol, to avoid bias from excluding participants who may have dropped out or not followed the protocol precisely. The PP analysis will only include participants who completed the study according to the protocol, providing a measure of efficacy under ideal conditions. This dual approach ensures the robustness and generalizability of the results. The statistical methods for primary and secondary outcomes are as follows.

### Primary outcome

The incidence rate of PHN between the two groups will be compared using the chi-square test. Relative risk (RR) and its 95% confidence interval will be calculated to evaluate the relative risk and effect of DXP with ropivacaine compared to antiviral treatment alone. Although randomization should control for most confounding factors, potential confounding factors such as sex age, baseline pain intensity, and comorbidities will be considered and controlled using multivariate logistic regression analysis.We will assess multicollinearity and select the most relevant predictors using methods like stepwise regression.

### Secondary outcomes

For quantitative data (e.g., NRS-11, SF-12, PSQI, and inflammatory markers), t-tests or Mann-Whitney U tests will be used depending on the data distribution characteristics to compare differences between the two groups. For categorical outcomes (e.g., complication rates), chi-square tests or Fisher’s exact tests will be used. Repeated measures ANOVA or mixed models will be employed to analyze time effects and group effects for continuous repeated measures data (e.g., quality of life scores at different time points post-treatment).

### Ethics and dissemination

This study adheres to the 2013 SPIRIT Statement and the Declaration of Helsinki and has been approved by the Ethics Committee of the Second Affiliated Hospital of Guangxi Medical University (Approval Number: 2024-KY(0505)). All participants must sign an informed consent form before enrollment, detailing the study’s purpose, procedures, potential risks and benefits, and privacy protection measures. This clinical trial is registered on the International Clinical Trials Registry Platform at https://www.chictr.org.cn/index.html (Registration Number: ChiCTR2400087273, Registration Date: 2024-07–24). The results will be disseminated through scientific journals and conferences.

## Discussion

The proposed study aims to investigate the efficacy and safety of ultrasound-guided thoracic paravertebral injection of DXP combined with ropivacaine in treating thoracic ZAP. Given the significant burden of ZAP and PHN on patients' quality of life, this study is of critical importance.

### Strengths and innovations

The innovative aspect of this study lies in its use of DXP, a liposomal formulation of dexamethasone, known for its high lipophilicity and prolonged duration of action. DXP’s pharmacokinetics allow it to be primarily distributed at sites of inflammation, gradually releasing the active compound, which enhances its anti-inflammatory efficacy and minimizes side effects compared to free dexamethasone (Lorscheider et al., 2019). This formulation, combined with ropivacaine, a sodium channel blocker with a long duration of action and small impact on motor function, offers a promising therapeutic approach for managing ZAP (Kaufman et al., 2005; Dresner et al., 2000; Hauritz et al., 2018).

TPVB has shown efficacy in alleviating ZAP and reducing the incidence of PHN. However, single injections are less effective, often requiring multiple injections for optimal effect (Ma et al., 2022; Abdelwahab et al., 2022; Deng et al., 2023; Kim et al., 2017) The proposed study hypothesizes that a single injection of DXP combined with ropivacaine can reduce the incidence of PHN and effectively alleviate ZAP, minimizing the need for repeated injections and thereby reducing the treatment burden on patients and healthcare systems.

### Comprehensive evaluation

The study’s design, which includes both immediate and follow-up assessments, allows for a comprehensive evaluation of both short-term and long-term outcomes. The primary outcome is the incidence rate of PHN, defined as an NRS-11 score of ≥4 3 months after rash onset. Secondary outcomes include reductions in pain intensity, improvements in sleep quality (using the Pittsburgh Sleep Quality Index), enhancements in quality of life (using the SF-12), decreases in weekly analgesic consumption, and changes in levels of inflammatory markers (CRP, TNF-α, IL-6). If successful, this study could provide a new, effective treatment option for managing ZAP and preventing PHN, significantly improving patient outcomes.

### Challenges and limitations

There are several potential challenges and limitations to this study. One challenge is ensuring patient adherence to the follow-up schedule, which is crucial for assessing long-term outcomes. To mitigate this, we will implement reminders and provide support to patients throughout the study period. Another limitation is the open-label design, which, while necessary due to the nature of the interventions, may introduce performance bias. We will minimize this by blinding the endpoint assessors and using standardized procedures for all assessments.

Additionally, this is a single-center study, which may limit the generalizability of the results. However, the single-center design allows for a higher level of control and standardization, potentially leading to more reliable and consistent data. Future multicenter studies would be necessary to confirm the findings and ensure broader applicability.

Another limitation is the inclusion of patients over 50 years old, which may not fully represent the broader population affected by herpes zoster. While this age group is more commonly associated with a higher incidence of herpes zoster and postherpetic neuralgia, future studies could consider including younger populations to determine if the findings are applicable to a wider age range.

## Conclusion

In conclusion, this study aims to provide valuable insights into the management of thoracic ZAP. By evaluating the efficacy and safety of DXP combined with ropivacaine, the study could pave the way for a new standard of care in treating ZAP and preventing PHN. The potential benefits of prolonged pain relief with fewer injections highlight the importance of this research in improving patient care and outcomes.

## Trial status

The protocol has been approved by the Second Affiliated Hospital of Guangxi Medical University. We plan to start recruiting trial subjects on 1 August 2024, and expect the study to last for 3 years to reach the target of 254 participants.
